# Migration in unicompartmental knee arthroplasty with the Persona Partial Knee: a cohort study of 26 patients using radiostereometry with 60 months of follow-up

**DOI:** 10.2340/17453674.2025.44995

**Published:** 2026-01-03

**Authors:** Jantsje H PASMA, Brechtje HESSELING, Nicole DE ESCH, Hennie VERBURG, Dieu D NIESTEN, Nina M C MATHIJSSEN

**Affiliations:** 1 Reinier Haga Orthopedisch Centrum, Zoetermeer; 2 Department of Orthopedics, Reinier de Graaf Hospital, Delft, The Netherlands

## Abstract

**Background and purpose:**

Migration is an early sign of loosening. We investigated the migration and stability of the cemented Persona Partial Knee (PPK, Zimmer Biomet, Warsaw, IN, USA), for both the femoral and tibial component, and evaluated the clinical results at 5 years’ follow-up.

**Methods:**

In this prospective cohort study, primary cemented PPKs were implanted. Migration of the tibial and femoral component at 5 years postoperatively was calculated using model-based radiostereometric analysis (mRSA) in terms of translations and rotations. To evaluate the clinical results, a clinical examination was performed using the Knee Society Score (KSS), and PROMs (NRS pain, KOOS-PS, OKS, EQ-5D) were registered.

**Results:**

26 patients were included. At 5 years postoperatively, we found low migration of both the tibial and femoral component, namely a translation of < 0.21 mm and rotation of < 0.75° in all directions for both components. Compared with 2 years’ follow-up, the tibial components showed an increased total translation and total rotation at 5 years. The femoral components showed stable migration compared with 2 years’ follow-up. The KSS decreased between 2 and 5 years, while the PROMs remained stable between 2 and 5 years’ follow-up.

**Conclusion:**

The PPK showed low migration of both the tibial and femoral components at 5 years’ follow-up. The femoral component was stable between 2 and 5 years, while the tibial component still migrated.

Patients with osteoarthritis limited to 1 compartment of the knee are suitable for treatment with a unicompartmental knee arthroplasty (UKA). Several registries have shown increasing proportions [1,2]. A UKA has several advantages over a total knee arthroplasty (TKA), such as shorter operative time, less invasive, less blood loss, fewer complications, and faster recovery [3-5]. Therefore, Dutch guidelines recommend the use of UKA in case of end-stage unicompartmental osteoarthritis of the knee, both for medial and lateral osteoarthritis [6]. However, a UKA is relatively more often revised compared with a TKA. In cemented UKA, one of the main reasons for revision is aseptic loosening [7,8]. Migration at an early stage is a warning for aseptic loosening [9].

Previous studies investigated the migration of both cementless and cemented medial UKA using model-based radiostereophotogrammetric analysis (mRSA) [10], a highly accurate and 3-dimensional method to measure the motion between a prosthesis and the host bone [9]. They showed low migration of cemented tibial components at 2 years after surgery [10]. However, 4–30% of the patients showed continuous migration of the tibial component between 1 and 2 years after surgery, which might result in a higher risk of revision [11-15]. In the mid-term, Mosegaard et al. showed low migration of the femoral component of medial UKA at 5 years, but significant total translation and rotation [16]. Campi et al. showed no significant migration of the tibial component of medial UKA between 2 and 5 years after surgery [17].

In our previous study, we reported the migration of the Persona Partial Knee (PPK, Zimmer Biomet, Warsaw, IN, USA), a cemented medial fixed-bearing UKA system, during 2 years’ follow-up [18]. The PPK is a relative new implant, whose design is based on the anatomical shape of the medial and lateral condyles. No information is available on the mid-term RSA results of this implant. We investigated the migration of the PPK, for both the tibial and femoral components, and the clinical outcomes up to 5 years after surgery.

## Methods

### Study design

This study is reported according to the STROBE reporting guideline [19].

This is a prospective study including patients with knee osteoarthritis. The 2 years’ results of this study have been reported previously [18]. Patients were included from April 2017 till May 2018 at the Reinier de Graaf Hospital, Delft, the Netherlands. Patients were followed during a period of 5 years. Outcome measures were obtained 60 months (±3 weeks) after surgery, in addition to the measurements preoperatively, direct postoperatively, and at 6 weeks (±1 week), 6 months (±1 week), 12 months (±2 weeks), and 24 months (±3 weeks) as described previously.

### Participants

Patients were considered for inclusion when indicated for a UKA, namely in the case of painful and/or disabling knee joints limited to the medial tibiofemoral compartment of the knee, due to non-inflammatory degenerative joint disease, traumatic arthritis, previous tibial condyle or plateau fractures, varus deformities, or revision of previous knee surgeries (except previous UKA). Patients were excluded in the case of an infection, rheumatoid arthritis or other inflammatory joint disease, neuromuscular diseases, a sensitivity or allergy to one or more materials of the prosthesis, or in the case of a revision UKA. Patients had to be able to speak and write Dutch. As the RSA guidelines indicate a low number of participants are needed to achieve a representative outcome measure, the sample size was set at 25 patients [20].

### Implants and surgery

The PPK is a cemented fixed-bearing medial UKA. The femoral component is made of a Co-Cr-Mo alloy, the tibial baseplate of a titanium alloy (Ti-6Al-4V), and the tibial bearing consists of vitamin-E stabilized highly crosslinked polyethylene (VEHXPE). The design is based on morphology of the global population, which might result in a more accurate, personalized, and anatomical fit. Both components were cemented with Optipac 40 Refobacin Bone Cement R (Zimmer Biomet, Warsaw, IN, USA).

The patients were admitted on the day of the surgery and received spinal anesthesia or spinal anesthesia combined with general anesthesia. The surgeries were performed by 2 surgeons (HV and DDN, 19 and 7, respectively) without navigation or other computer-assisted instruments. During surgery, 6–9 tantalum marker beads (1.0 mm in diameter) were inserted in the femur and 6–9 tantalum marker beads in the tibia after bone preparation. After surgery, a standardized protocol was used for pain medication. Patients began mobilization of the day of surgery. Patients were discharged contingent upon (i) their being able to perform some daily activities and (ii) the pain was relieved sufficiently.

### Outcome measures

Outcome measures were obtained 60 months (±3 weeks) after surgery. Clinical examination was performed in line with the clinical part of the Knee Society Score (KSS), consisting of the range of motion, alignment, and stability of the medial and lateral collateral ligaments [21]. Standard radiographs consisted of anteroposterior and lateral view radiographs.

#### Patient-reported outcome measures (PROMs)

PROMs were used to investigate the clinical and functional outcomes. The Knee injury and Osteoarthritis Outcome Score (KOOS-PS) [22], Numeric Rating Scale for pain (NRS pain) [23], Oxford Knee Score (OKS) [24], and EuroQol-5D (EQ-5D-5L) [25] were filled in by the patient at each follow-up moment. The KOOS-PS is a measure of physical function for the knee (range 0–100, higher score indicates more problems). NRS pain scores the pain at rest and during movement (range 0–10, higher score indicates more pain). The OKS addresses knee function (range 0–48, higher score indicates better function). EQ-5D-5L measures the quality of life by both 5 questions concerning the perceived quality of life (range –0.446 to 1, higher score indicates better quality of life) and a VAS score of overall health (range 0–100, higher score indicates better overall health).

#### Model-based radiostereophotogrammetric analysis

To perform mRSA, stereoradiographs were obtained in supine position at 5 years’ follow-up with a standardized RSA set-up [18]. Model-based RSA software (version 4.2, Medis Specials, LUMC, Leiden, the Netherlands) was used for the analysis following the consistent-marker method and according to the International Organization for Standardization (ISO) and guidelines [9].

Implant migration of both the femoral and tibial component was calculated using all 6 stereoradiographs with the direct postoperative stereoradiograph as reference. Translations were calculated using the center of gravity of the bone markers as the reference object and the 3-dimensional model of the implant as migrating object. Data was normalized to presentation for the right knee; translations were expressed in mm along the x-axis (+ medial / – lateral), y-axis (+ cranial/proximal / – caudal/distal), and z-axis (+ anterior / – posterior) and rotations were expressed in degrees about the x-axis (+ anterior tilt / – posterior tilt), y-axis (+ internal/endorotation / – external/exorotation), and z-axis (+ adduction / – abduction) in a globally aligned coordinate system located in the center of the implant model with the y-axis parallel to the anatomical axis of the leg. Furthermore, total translation (i.e., the Euclidian distance between the initial position of the center of gravity of a component and its position at follow-up), total rotation (calculated in the same way), and the maximum total point motion (MTPM, i.e., the Euclidian distance between the initial position and the position at follow-up of the point that moved most) were calculated.

A minimum of 3 bone markers are needed to analyze the rotations. Markers were excluded from analysis in the case of instability (mean error > 0.35 mm). As a marker-consistent analysis was used, not all the same markers might be used compared with our previous analysis. To include stereoradiographs with < 3 bone markers, a mean marker model (MC model) was made. In the case of an MC model, all available markers at the postoperative stereoradiographs and all follow-up stereoradiographs were used. As 1 follow-up stereoradiograph was added, this might result in small differences in migration compared with the previously published results. Subjects were excluded from analysis in the case of < 3 bone markers, a condition number of > 120 and/or a rigid body fitting error of > 0.35 mm.

Double examinations were performed at 1-year follow-up to calculate the bias, precision, and precision interval, defined as the mean, SD, and 1.96 x SD, respectively, of the migration between the 2 examinations with the first examination as a reference. In the case of non-normality only the SD and mean were calculated. In addition to the precision intervals for the separate translations and rotations calculated in our previous study [18], we calculated the precision of the MTPM, total translation, and total rotation in the current study (Table 1). Values within the precision interval might be measurement errors and therefore not real migrations.

**Table 1:** Precision results of double examinations of the femoral and tibial component

Migration	Femoral component (n = 11)		Tibial component (n = 17)	
	Mean (SD)	PI	Mean (SD)	PI
Translation, mm				
X (+ medial/ – lateral)	0.013 (0.12)	0.23	–0.019 (0.074)	0.15
Y (+ proximal/ – distal)	–0.012 (0.038)	0.074	–0.010 (0.057)	0.11
Z (+ anterior/ – posterior)	–0.002 (0.22)	0.43	0.006 (0.10)	0.31
Rotation, degrees				
X (+ anterior tilt/ – posterior tilt)	0.046 (0.097)	0.19	0.072 (0.40)	0.79
Y (+ endorotation/ – exorotation)	–0.036 (0.32)	0.62	–0.035 (0.25)	0.48
Z (+ adduction/ – abduction)	–0.060 (0.15)	0.30	0.094 (0.29)	0.57
Total translation, mm	0.17 (0.18)		0.13 (0.13)	
Total rotation, degrees	0.28 (0.22)		0.44 (0.34)	
MTPM, mm	0.26 (0.26)		0.28 (0.20)	

PI: precision interval.

### Statistics

All data was checked for normality. In the case of a normal distribution, data was presented as mean with SD. Otherwise, median with IQR or count was presented. Non-normal migration data is presented by mean, median, and range according to Kaptein et al. and Valstar et al. [9,20].

PROMs at 5 years’ follow-up were compared with PROMs at 2 years’ follow-up. Migration in each direction (i.e., individual testing) at 5 years’ follow-up was compared with zero (i.e., direct postoperative) to investigate whether significant migration occurred. Next, the migration was compared with migration at 2 years’ follow-up to investigate whether there was significant migration between 2 and 5 years. Because of individual testing, no correction for multiple testing was applied. All analyses were performed using linear mixed models, taking into account the longitudinal nature of the measurement per patient and missing values. Statistical significance was assumed at P < 0.05. All data was analyzed using SPSS version 28.0.0.0 (IBM Corp Armonk, NY, USA).

### Ethics, data sharing plan, funding, use of AI, and disclosures

The study was reviewed and approved by the Ethics Medical Committee (METC Zuidwest Holland, METC-nr 16-031, NL60028.098.16) and performed in accordance with the Declaration of Helsinki (2013). The study is registered in the Dutch Trial Register (NL-OMON20822). All participants gave written informed consent.

Data cannot be shared. The research department receives grants from Zimmer Biomet and Stryker to perform clinical studies. No AI was used in performing the study, analyzing the radiographs and results, and writing this article.

Complete disclosure of interest forms according to ICMJE are available on the article page, doi: 10.2340/17453674.2025.44995

## Results

**Figure 1:**
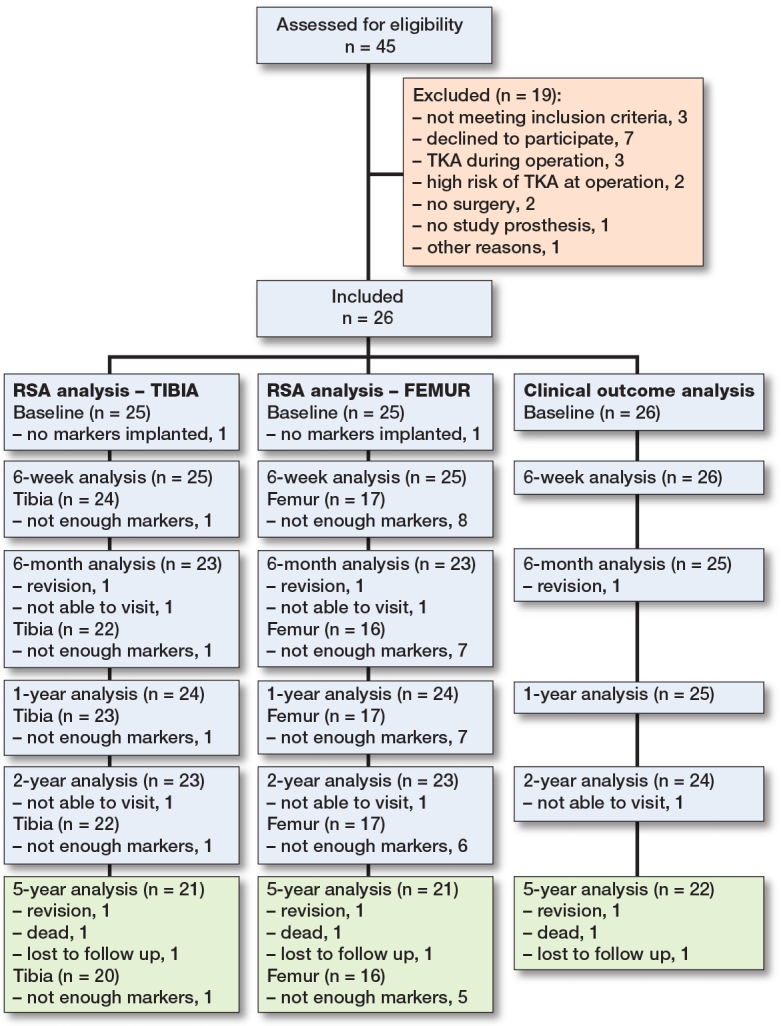
Flow diagram of patient inclusion and follow-up.

In total, 26 patients were included from April 2017 till May 2018 at the Reinier de Graaf Hospital, Delft, the Netherlands (Figure 1). In this period, 45 patients underwent a UKA in the Reinier de Graaf Hospital; 19 patients were excluded, for several reasons (Figure 1).

Table 2 describes the patient characteristics and surgical details of the 26 included patients. The mean age was 63.5 years (standard deviation [SD] 7.4) and 15/26 patients were female. The median duration of complaints was 2.5 years (interquartile range [IQR] 1.0–5.0).

**Table 2:** Patient characteristics and surgical details. Values are presented as mean with standard deviation, count, or as specified

Factor	N = 26
Age, years	63.5 (7.4)
Female sex	15
Height, m	1.73 (0.12)
Weight, kg	89.8 (9.8)
Body mass index	30.2 (4.0)
Right side	15
ASA score	
I	7
II	15
III	4
Duration complaints, years **^a^**	2.5 (1–5)
Previous surgery	18
Operation time, minutes **^a^**	60 (54–68)
Duration of anesthesia, minutes **^a^**	89 (83–96)

ASA = American Society of Anesthesiologists Physical Status classification

a median (IQR)

### Radiostereophotogrammetric analysis

Table 3 presents the features of the analyses, namely the number of markers used, the mean error of rigid-body fitting and the condition number. A Supplementary Table with all migration values is available on the article homepage.

**Table 3:** Features of the analyses for both the femoral and the tibial component. Values are presented as mean with standard deviation

Item	Femoral component					Tibial component				
	6 weeks	6 months	12 months	24 months	60 months	6 weeks	6 months	12 months	24 months	60 months
	n = 17	n = 16	n = 17	n = 16	n =16	n = 24	n = 22	n = 23	n = 22	n = 20
Markers, n	4.8 (1.2)	4.7 (1.3)	4.6 (1.3)	4.8 (1.2)	4.8 (1.2)	6.5 (1.4)	6.5 (1.4)	6.5 (1.4)	6.6 (1.3)	6.6 (1.4)
Condition number	64 (23)	64 (24)	66 (25)	68 (24)	68 (24)	51 (17)	51 (17)	52 (17)	52 (17)	52 (18)
Mean error of rigid-body fitting	0.05 (0.06)	0.05 (0.07)	0.05 (0.07)	0.05 (0.08)	0.05 (0.08)	0.12 (0.06)	0.15 (0.08)	0.16 (0.07)	0.17 (0.07)	0.19 (0.08)

**Figure 2:**
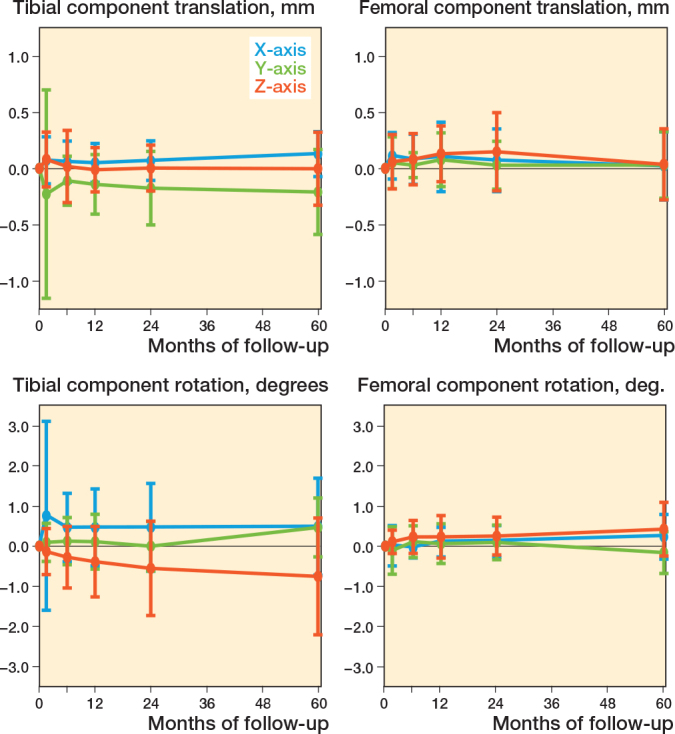
Migration results for the tibial and femoral component during 60 months presented by the translation along the x-axis (+ medial/ – lateral) (blue), y-axis (+ proximal/ – distal) (green), and z-axis (+ anterior/ – posterior) (orange), and rotations about the x-axis (+ anterior tilt/ – posterior tilt) (blue), y-axis (+ endorotation/ – exorotation) (green), and z-axis (+ adduction/ – abduction) (orange). Data is presented as mean with 1 standard deviation.

**Figure 3:**
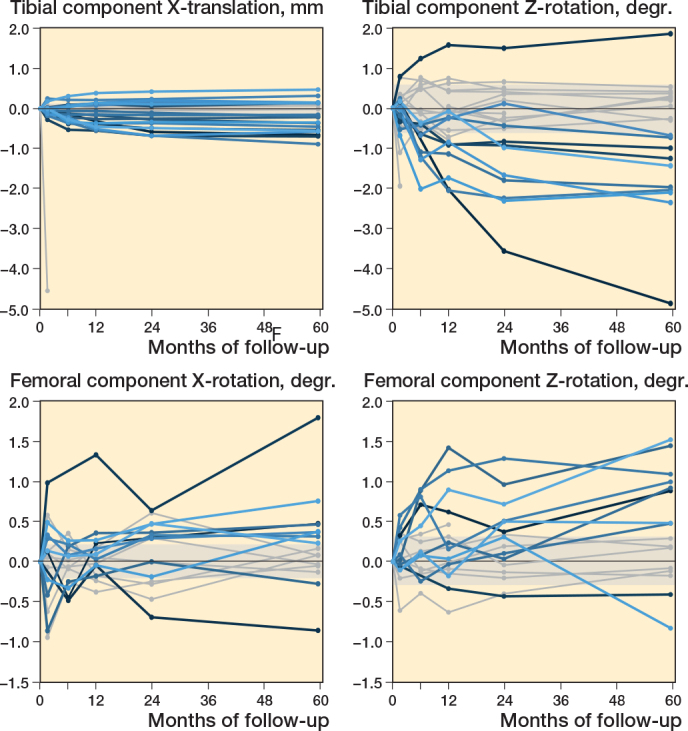
Spaghetti plots of the translation along the y-axis (+ proximal/ – distal) and the rotation about the z-axis (+ adduction/ – abduction) for the tibial component, and of the rotation about the x-axis (+ anterior tilt/ – posterior tilt) and z-axis (+ adduction/ – abduction) of the femoral component during 60 months of follow-up. Patients with migration outside the precision interval at 60 months’ follow-up are indicated in blue. The shaded areas indicate the precision interval.

**Figure 4:**
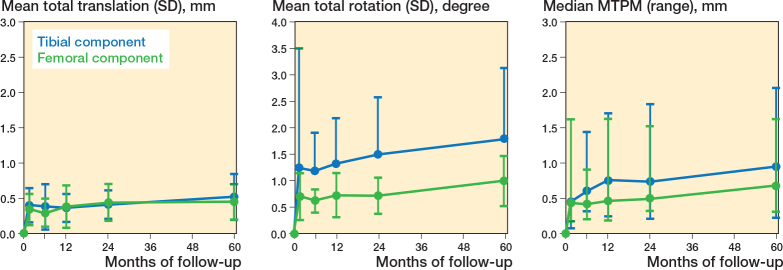
Total translation, total rotation, and maximum total point motion (MTPM) for the tibial (blue) and femoral (green) component during 60 months. Data is presented as mean with 1 standard deviation. The MTPM is presented as median with range.

The femoral component shows a significantly positive rotation along the x-axis (anterior tilt) and z-axis (adduction) at 5 years’ follow-up (mean (SD) of 0.24° (0.56) and 0.43° (0.67), respectively, compared with baseline (P < 0.03), which was outside the precision interval and was therefore indicated as a real migration (i.e., no measurement error) (Figures 2 and 3). These rotations were stable between 2 and 5 years (P > 0.2). Compared with the 2 years’ follow-up, the femoral component shows stable translations along the x-axis (medial translation) and y-axis (proximal translation) and stable rotation about the y-axis (endorotation) between 2 and 5 years’ follow-up, which were within the precision interval (P > 0.08). The femoral component only significantly translated back to zero along the z-axis (anterior translation) at 5 years’ follow-up (P = 0.04). However, this was within the precision interval. Together this resulted in a stable MTPM (median 0.69, range 0.32–1.62 mm at 5 years’ follow-up) (P = 0.2) and total translation (P = 0.8) between 2 and 5 years after surgery, but a significantly increased total rotation between 2 and 5 years (P = 0.02) (Figure 4).

All translations and rotations of the tibial component, except the negative translation along the z-axis (posterior translation), were significantly changed at 5 years’ follow-up compared with baseline (P < 0.04) (see Figure 2). Only the negative translation along the y-axis (distal translation) and the negative rotation about the z-axis (abduction) were outside the precision interval at 5 years’ follow-up (mean –0.21 [SD 0.38] mm and –0.75° [SD 1.46], respectively), indicating a real migration (see Figure 3). Between 2 and 5 years’ follow-up, these migrations were stable (P > 0.2). Only the positive rotation about the y-axis (endorotation) of the tibial component significantly increased at 5 years’ follow-up compared with 2 years’ follow-up (P < 0.001). However, this rotation was still within the precision interval. Together this resulted in a significantly increased MTPM (median 0.96, range 0.24–2.6 mm at 5 years’ follow-up) (P = 0.02), total translation (P = 0.03), and total rotation (P = 0.03) between 2 and 5 years’ follow-up (see Figure 4).

### Clinical outcomes

The KSS worsened significantly after 5 years’ follow-up compared with 2 years’ follow-up (mean difference –17 [SD 24], MCID 9.7, CI 7.3–10.2 [26]) (P < 0.001), while the range of motion significantly improved (mean difference 4 [SD 8], MCID 12.8, CI 0.0–26.3 [27]) (P = 0.03) (Table 4). All PROMs, namely the NRS at rest and during movement, OKS, KOOS-PS, and EQ-5D-5L, were stable between 2 and 5 years’ follow-up (all P > 0.5) (Table 4).

**Table 4:** Clinical outcomes. Values are mean with standard deviation or as specified

Item	Preoperative	6 weeks	6 months	12 months	24 months	60 months
	n = 26	n = 26	n = 25	n = 25	n = 24	n = 22
KSS, points, median (IQR)	76 (61–85)	83 (72–91)	96 (89–99)	93 (84–100)	99 (85–100)	75 (68–78)
Range of motion, degrees	117 (19)	112 (12)	126 (6)	128 (5)	127 (7)	132 (8)
NRS (at rest), score	4.5 (2.9)	3.4 (2.9)	1.5 (1.6)	1.4 (1.9)	2.0 (2.4)	1.6 (2.5)
NRS (during movement), score	7.2 (1.7)	4.2 (3.0)	2.6 (1.9)	2.4 (2.4)	2.4 (2.6)	2.4 (3.0)
OKS, points	22 (8)	32 (10)	40 (7)	40 (6)	40 (10)	40 (10)
KOOS-PS, points	53 (19)	39 (18)	26 (16)	27 (12)	26 (16)	26 (21)
EQ-5D-5L, points, median (IQR)	0.38 (0.17–0.64)	0.54 (0.16–0.79)	0.81 (0.27–1.00)	0.81 (0.69–1.00)	0.84 (0.61–1.00)	0.90 (0.80–1.00)
EQ-5D-5L (VAS), score	67 (22)	77 (17)	75 (21)	79 (15)	73 (27)	75 (19)

### Complications

One revision of the tibial component occurred 15 weeks after surgery because of a fracture of the tibia following trauma. Between 2 and 5 years’ follow-up, 1 patient was revised 42 months after surgery because of loosening of the tibial component and 1 patient died 26 months after surgery for reasons unrelated to the prosthesis.

## Discussion

We aimed to investigate the migration of the cemented medial PPK, for both the femoral and tibial components, and evaluated the clinical results at 5 years’ follow-up. We found a low migration of both the femoral and tibial component during 5 years’ follow-up. Between 2 and 5 years’ follow-up the femoral component was stable, while the tibial component still migrated, which was represented by a stable MTPM of the femoral component and a significantly increased MTPM of the tibial component, respectively.

### Migration

**Figure 5:**
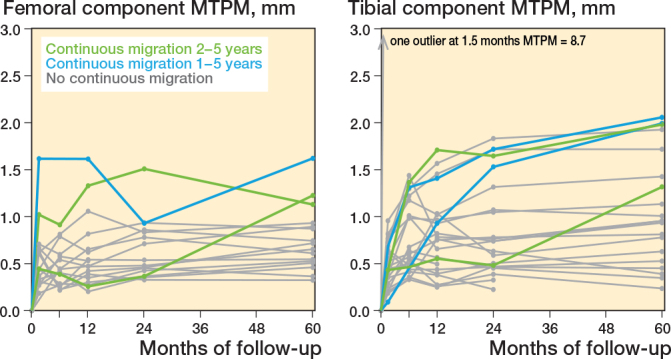
Spaghetti plots of the maximum total point motion (MTPM) for the tibial and femoral component during 60 months. Patients indicated in green show continuous migration between 2 and 5 years’ follow-up; patients indicated in blue show both continuous migration between 1 and 2 years’ and between 2 and 5 years’ follow-up.

Based on the MTPM, the migration of the femoral component at 5 years’ follow-up was low and does not differ from the migration at 2 years’ follow-up. This indicates that the femoral component is stable between 2 and 5 years’ follow-up. However, the total rotation of the femoral component does show a significant increase between 2 and 5 years, which might be due to the not statistically significant increase in rotation about the z-axis (adduction). This shows the importance of further following up the migration of the femoral component. The migration of the femoral component at 5 years’ follow-up is comparable to other studies. Mosegaard et al. and Campi et al. also showed low migration of the femoral component at 5 years’ follow-up and no significant migration between 2 and 5 years’ follow-up, but significant total translation and rotation [16,17]. However, Mosegaard et al. showed continuous migration in 12/72 patients, which was defined as an MTPM change from 2 to 5 years ≥ 0.3 mm [28]. In our study, we found a comparable percentage of patients with continuous migration, namely 3/16 patients. One of these patients also showed continuous migration between 1 and 2 years’ follow-up (defined as a difference in MTPM > 0.2 mm between 12 and 24 months postoperatively) (Figure 5).

The migration of the tibial component is small but continuous, as shown by the increased MTPM, total translation, and total rotation between 2 and 5 years’ follow-up. This is mainly due to the positive rotation about the y-axis (endorotation), the negative rotation about the z-axis (abduction), and the negative translation along the y-axis (distal translation). The rotation about the y-axis remains within the precision interval and therefore is not real migration. In contrast, both the rotation about the z-axis and the translation along the y-axis at 5 years’ follow-up fell outside the precision interval, which indicates these are real migrations. The migration of the tibial component at 5 years’ follow-up is comparable to the study by Campi et al. However, Campi et al. did not show significant migration between 2 and 5 years [17].

Our previous study described that 7 patients showed continuous migration of the tibial component between 1 and 2 years’ follow-up. In this study, in 4/20 patients we found continuous migration of the tibial components between 2 and 5 years’ follow-up. Of these patients, 2 patients also showed continuous migration between 1 and 2 years after surgery.

### Clinical outcomes

All PROMs showed a clinically relevant change compared with baseline and were stable between 2 and 5 years’ follow-up. The PROMs are comparable to other studies investigating the clinical outcomes after UKA [29-31]. The KSS decreased between 2 and 5 years’ follow-up. This decrease was higher than the minimal clinically relevant difference [26] and was mainly due to an increase in mediolateral and anteroposterior instability. Instability is a known failure in fixed-bearing UKA [32]. Longer follow-up may show the impact of this decrease on the revision rate. On the other hand, the range of motion shows a significant improvement between 2 and 5 years. This increase was smaller than the minimal clinically relevant difference [27]. However, a small number of patients were included in this study, which makes it difficult to draw conclusions from these findings. The KSS score at 5 years after surgery is still higher than the patient acceptable symptom state (PASS) thresholds at 2 and 10 years after UKA (85.5 and 70.5 respectively) as described by Goh et al. and Tan et al. [29,33].

### Migration and clinical outcomes

Previous studies show a relation between the migration and loosening of the tibial component in TKA, in which patients with the highest migration exhibited aseptic loosening, and defined thresholds to predict aseptic loosening [34-36]. These thresholds are neither available for the femoral component in TKA nor for UKA. Using the TKA thresholds of Pijls et al. [35], both the femoral and tibial component are at risk with a mean MTPM between 0.5 and 1.6 mm at 1-year follow-up. Based on the updated TKA thresholds of Puijk et al. [36], this cemented UKA is still at risk, with a mean MTPM between 0.3 and 1.1 mm at 6 months’ follow-up.

The femoral component is stable with a mean MTPM increase < 0.2 mm between 2 and 5 years, while the tibial component is unstable with a mean MTPM increase > 0.2 mm between 2 and 5 years. However, the thresholds of Pijls et al. are defined for migration until 2 years’ follow-up and do not give information regarding the thresholds of the migration between 2 and 5 years’ follow-up. Furthermore, Puijk et al. showed no relation between the mean migration between 1 and 2 years and the revision rate at 5, 10, or 15 years’ follow-up.

Using the TKA thresholds of Gudnason et al. [34], several patients show migration of the tibial component above the thresholds (6 patients > 0.8° rotation about the x-axis, 3 patients > –0.6 mm translation along the y-axis). One patient shows migration of the femoral component above the thresholds; however, this patient was already indicated as an outlier due to a measurement error. The thresholds of Gudnason et al. were defined for migration at 2 years’ follow-up and not for migration at 5 years’ follow-up. Further research is needed to gain more insight into whether these thresholds also hold for later time points.

### Strengths and limitations

Not all RSA radiographs could be analyzed to determine the migration of the femoral component as not enough markers were visible on the RSA radiographs. Furthermore, a relatively low number of participants were included in the study. This number is enough for RSA purposes but makes it challenging to draw conclusions concerning clinical performance and to relate the measured migration to clinical outcomes. Long-term follow-up is needed to gain more insight into the migration and to investigate the clinical outcomes. Therefore, we shall investigate the 10 years’ follow-up in the future.

### Conclusion

Both the femoral and tibial component of the PPK show low migration and good clinical results at mid-term follow-up. Only the migration of the tibial component continued between 2 and 5 years’ follow-up. Long-term results are needed to gain more insight into the migration patterns of UKA and to evaluate the risks of continuous migration.

### Supplementary data

The Supplementary Table is available on the article home-page, doi: 10.2340/17453674.2025.44995

## Supplementary Material



## Notes

HV, DDN, NMCM, and BH designed the study. HV, DDN, and NE informed and included patients. HV and DDN performed the surgeries. NE coordinated the follow-up visits. JHP analyzed the data and wrote the manuscript. All authors critically reviewed the manuscript and approved the manuscript for submission.

The authors would like to thank Rianne Oomen and Ian Blom for support in taking and analyzing RSA radiographs.

Handling co-editor: Per Henrik Randsborg

*Acta* thanks Stephan Maximilian Röhrl and Svend Erik Østgaard for help with peer review of this manuscript.
