# Supplementary material for: Migration in unicompartmental knee arthroplasty with the Persona Partial Knee: a cohort study of 26 patients using radiostereometry with 60 months of follow-up
Source: Acta Orthop. 2026 Jan 3;97:1–8. doi: 10.2340/17453674.2025.44995 (PMC12766172; doi:10.2340/17453674.2025.44995)
Supplement: Supplementary file 1 [file ActaO-97-44995-s1.pdf]

Supplementary material

Supplementary Table 1. Migration results of the femoral and tibial component. Data is presented as mean with standard deviation, except for the maximum total point motion (MTPM), which was not normally distributed. MTPM is presented by the mean, median, and range

	Femoral component					Tibial component				
	6 weeks<br>n = 17	6 months<br>n = 16	12 months<br>n = 17	24 months<br>n = 16	60 months<br>n =16	6 weeks<br>n = 24	6 months<br>n = 22	12 months<br>n = 23	24 months<br>n = 22	60 months<br>n = 20
Translation, mm										
X (+ medial/ – lateral)	0.112 (0.207)	0.081 (0.223)	0.102 (0.309)	0.072 (0.279)	0.022 (0.304)	0.072 (0.209)	0.060 (0.183)	0.049 (0.173)	0.069 (0.176)	0.127 (0.200)
Y (+ proximal/ –distal)	0.050 (0.230)	0.029 (0.111)	0.077 (0.239)	0.028 (0.213)	0.030 (0.296)	-0.229 (0.927)	-0.110 (0.218)	-0.142 (0.265)	-0.176 (0.327)	-0.210 (0.378)
Z (+ anterior/ – posterior)	0.059 (0.242)	0.082 (0.228)	0.130 (0.248)	0.148 (0.348)	0.036 (0.316)	0.079 (0.244)	0.017 (0.321)	-0.012 (0.197)	0.003 (0.204)	-0.003 (0.325)
Total	0.34 (0.22)	0.29 (0.20)	0.38 (0.30)	0.44 (0.26)	0.45 (0.25)	0.40 (0.93)	0.38 (0.22)	0.36 (0.17)	0.41 (0.21)	0.52 (0.26)
Rotation, deg										
X (+ anterior / – posterior tilt)	0.012 (0.503)	-0.039 (0.255)	0.102 (0.370)	0.119 (0.398)	0.236 (0.557)	0.760 (2.356)	0.466 (0.854)	0.470 (0.960)	0.472 (1.095)	0.489 (1.209)
Y (+ endo-/ – exorotation)	-0.105 (0.592)	0.114 (0.396)	0.066 (0.495)	0.097 (0.432)	-0.158 (0.521)	0.097 (0.475)	0.128 (0.588)	0.114 (0.683)	0.002 (0.620)	0.469 (0.734)
Z (+ adduction/ – abduction)	0.111 (0.291)	0.233 (0.411)	0.235 (0.531)	0.255 (0.471)	0.426 (0.670)	-0.132 (0.574)	-0.273 (0.768)	-0.384 (0.879)	-0.551 (1.176)	-0.752 (1.456)
Total	0.70 (0.44)	0.62 (0.22)	0.73 (0.41)	0.72 (0.33)	1.00 (0.48)	1.25 (2.26)	1.19 (0.71)	1.33 (0.85)	1.50 (1.08)	1.79 (1.35)
MTPM, mm, mean	0.545	0.473	0.606	0.645	0.758	0.825	0.779	0.800	0.884	1.080
median	0.438	0.426	0.472	0.508	0.685	0.456	0.619	0.762	0.740	0.956
range	(0.18–1.62)	(0.22–0.91)	(0.20–1.62)	(0.33–1.51)	(0.32–1.62)	(0.09–8.74)	(0.33–1.44)	(0.25–1.71)	(0.22–1.83)	(0.24–2.06)
